# Genome drafts of *Lotmaria passim* strains C2 and C3 isolated from honey bees in Spain

**DOI:** 10.1128/mra.00642-24

**Published:** 2024-11-27

**Authors:** José Luis Ruiz, Arturo Marin, Jessica Carreira de Paula, Tamara Gómez-Moracho, Pedro García Olmedo, Eduardo Andrés-León, Jennifer Solano Parada, Francisco Orantes Bermejo, Antonio Osuna, Luis Miguel de Pablos

**Affiliations:** 1 Bioinformatics Unit, Institute of Parasitology and Biomedicine “López-Neyra” (IPBLN), CSIC, Granada, Spain; 2 Omics Bioinformatics S.L., Granada, Spain; 3 Department of Parasitology, Biochemical and Molecular Parasitology Group CTS-183, University of Granada, Granada, Spain; 4 Institute of Biotechnology, University of Granada, Granada, Spain; 5 Apinevada S.L., Granada, Spain; Indiana University, Bloomington, Bloomington, Indiana, USA

**Keywords:** bee, *Lotmaria*, genome, *Trypanosoma*, *Crithidia*, parasite

## Abstract

*Lotmaria passim* is a highly prevalent parasite of honey bees. Herein is reported the draft genome sequences of *L. passim* C2 and C3 strains of 27.15 Mbp and 26.94 Mbp, respectively. The genomes were sequenced using Illumina MiSeq platform and will allow for further comparative and functional genomics studies.

## ANNOUNCEMENT

Bee parasites are one of the main drivers for bee colony losses worldwide (1). Among the current threats, different species of parasites belonging to the family Trypanosomatidae (Euglenozoa, Kinetoplastea) have been found infecting honey bees and wild bees (2–5). Within trypanosomatids, *Lotmaria passim* is the most prevalent in bee colonies worldwide (2, 6–8). This parasite has been associated with winter colony losses (6), and it produces an increase in honey bee mortality in experimental infections (5, 9, 10). The life cycle of the parasite includes a differentiation program from unicellular flagellar state to multicellular biofilm microcolonies found in the hindgut of their hosts (11). Thus, *L. passim* has risen as an informative biological model for parasite-host interaction studies. Since reverse genetics functional genomics tools are available for these parasites, it is necessary to generate new improved reference draft genomes for their study and genomic manipulation.

Thus far, only two genome drafts (Bioproject IDs: PRJNA78249, PRJNA319529) have been deposited at NCBI. Apart from those, a recent chromosome-level genome of *L. passim* (Bioproject ID: PRJNA1049372) has been resolved into 31 contigs in its nuclear genome together with a kinetoplast genome composed by a maxicircle and 30 minicircle sequences (12). In this work, we generated two new isolates called *L. passim* C2 and *L. passim* C3 from a honey bee foraging at *Lavandula angustifolia* in May 2019 at Granada city (Spain, 37°10′54.3″N; 3°36′37.1″W) and from an infected colony at Béznar (Spain, 36°55′25.1″N; 3°31′42.9″W). The parasites were isolated as it was previously described (10), axenically cultured in modified Brain Heart Infusion Triptose (BHT) media up to midlog phase, and identified by isolating gDNA by using the standard phenol-chloroform DNA extraction method and sequencing SSU rRNA sequence with the oligonucleotides S-762 and S-763, as previously described (10, 13).

Once the gDNA was isolated, the Genomics Unit at IPBLN-CSIC performed library preparation using the Covaris M220 for sonication, the NEBNext Ultra II DNA Library Preparation Kit for Illumina, and the Bioanalyzer High Sensitivity DNA assay for quality control. The libraries were sequenced by Illumina MiSeq V2 paired-end 250 bp, yielding an output of 9.74 Gbp and a sequencing depth of ~175×. For all software, default parameters were used except where otherwise noted. For each isolate, around 18–20 million sequencing reads were assembled using MaSuRCA v4.1.0 (14) (JF_SIZE = 6.562E^8^, and 453/122 and 428/112 as fragment mean and standard deviation were used as parameters for C2 and C3 strains, respectively). For the quality control and the polishing of the *de novo* draft assemblies, the pipeline ILRA v1.4.2 (15) was used. The automatic finishing of the genome included steps such as filtering of contigs based on a size threshold, merging of overlapping contigs, iterative polishing (*n* = 3) by iCORN2 (16), decontamination by Kraken2 (17) leveraging the full “nt” database (18) and by KrakenTools (19) to extract the sequences assigned to the subfamily Leishmaniinae (taxid: 1286322), decontamination by MegaBLAST (20) using several databases conforming to the Foreign Contamination Screen by NCBI (21), or quality control by BUSCO (22) and QUAST (23). Annotation was performed with Companion (web server November 2022) (24), using *Leptomonas pyrrhocoris* H10 as the closest reference genome and parameters by default, except for 0.2 as the AUGUSTUS score threshold. Table 1 contains further details for all sequences, including some of the improvements by ILRA and the final assembly statistics.

**TABLE 1:** Overview of the *Lotmaria passim* sequencing datasets presented in this study, together with the statistics of the assembly by MaSuRCA, the step-by-step corrections by ILRA, and the annotation by Companion

*Lotmaria passim* strain			C2	C3
Sequencing statistics	Sequencing reads (million)		650	623
	Average size (bp)		19.8	17.9
Assembly (MaSuRCA, pre-ILRA correction)	Assembly size (Mbp/*n* contigs)		29.54/671	29.81/698
	N50 (kbp)/L50 (*n* contigs)		183.16/40	179.04/50
Post-ILRA correction	Discarded contigs	Threshold of <5 kbp	341	347
		Merging due to overlapping	32	21
		Labeled as contamination (Kraken2)	52	74
	Assembly size (Mbp/*n* contigs)		27.15/257	26.94/252
	N50 (kbp)/L50 (*n* contigs)		216.55/34	194.62/42
	Gaps		46	57
	GC content (%)		54.23	54.19
	Completeness (BUSCO euglenozoa_odb10, %)		96.2	99.10
	Sequencing depth (×)		182	166
Companion annotation	# Predicted genes/coding/with function		8,291/8,180/5,269	8,261/8,153/393
	# Predicted pseudogenes		412	393

The final assembly sizes were 27.15 Mbp comprising 257 contigs with N50 216.5 kbp for *L. passim* C2 and 26.94 Mbp comprising 252 contigs with N50 194.6 kbp for *L. passim* C3. A contig was labeled as mitochondria with ~99% identity in both cases. Further details are described in Table 1. These results could be used for comparative analysis with strains which were previously used as models of cell differentiation *in vitro* and *in vivo* (11) and set the stage for future functional genomics studies in *L. passim*.

## Data Availability

This whole genome sequencing and assembly of *L. passim* C2 and C3 strains have been deposited in GenBank under the accession numbers GCA_030247965 and GCA_030247975, respectively. The bioproject PRJNA863431 contains the raw data. The protein and genome annotation, together with the genome assemblies and raw data have been deposited in Zenodo under DOI 10.5281/zenodo.13906989.
